# Vaccination Coverage at Birth in Brazil: Spatial and Temporal Trends in the Impact of COVID-19 on Uptake of BCG and Hepatitis B Vaccines

**DOI:** 10.3390/vaccines12121434

**Published:** 2024-12-20

**Authors:** Yan Mathias Alves, Thaís Zamboni Berra, Reginaldo Bazon Vaz Tavares, Nathalia Zini, Quézia Rosa Ferreira, Licia Kellen de Almeida Andrade, Ariela Fehr Tártaro, Maria Eduarda Pagano Pelodan, Beatriz Fornaziero Vigato, Beatriz Kuroda Silveira, Ana Luiza Brasileiro Nato Marques Assumpção, Marcela Antunes Paschoal Popolin, Patricia Abrahão Curvo, Simone Protti-Zanatta, Maria Del Pilar Serrano-Gallardo, Ricardo Alexandre Arcêncio, Pedro Fredemir Palha, Jaqueline Garcia de Almeida Ballestero

**Affiliations:** 1 Department of Maternal-Infant and Public Health Nursing, Ribeirão Preto College of Nursing, University of São Paulo, Ribeirão Preto 14040-902, São Paulo, Brazil; thaiszamboni@live.com (T.Z.B.); reginaldobazon@usp.br (R.B.V.T.); nathalia_zini@hotmail.com (N.Z.); quezia@usp.br (Q.R.F.); liciaandrade@usp.br (L.K.d.A.A.); ariela.ft@usp.br (A.F.T.); mpelodan@usp.br (M.E.P.P.); beatrizfornazierovigato@usp.br (B.F.V.); kurodabeatriz@usp.br (B.K.S.); analuizaassumpcao@usp.br (A.L.B.N.M.A.); patricia@eerp.usp.br (P.A.C.); ricardo@eerp.usp.br (R.A.A.); palha@eerp.usp.br (P.F.P.); 2 Nursing Department, Federal University of Tocantins, Palmas 77001-090, Tocantins, Brazil; marcelappopolin@gmail.com; 3 Nursing Department, Federal University of São Carlos, São Carlos 13565-905, São Paulo, Brazil; simoneprotti@ufscar.br; 4 Nursing Department, Faculty of Medicine, Autonomous University of Madrid, 28029 Madrid, Spain; pilar.serrano@uam.es

**Keywords:** vaccine hesitancy, vaccination strategies, BCG, hepatitis B, epidemiology

## Abstract

Introduction: Vaccines are a significant public health achievement, which are crucial for child survival and disease control globally. In Brazil, the National Immunization Program (PNI) manages vaccination schedules, including essential vaccines like BCG and Hepatitis B, administered at birth. Despite achieving over 95% coverage for years, vaccination rates have declined since 2016, a trend exacerbated by the COVID-19 pandemic. This study aims to analyze spatial and temporal trends in BCG and Hepatitis B vaccination coverage at birth, identify areas with spatial variation in these trends, classify the identified trends, and investigate the pandemic’s impact on vaccination adherence. Methods: This is an ecological study with real-world data from Brazil, focusing on vaccination coverage from 2014 to 2023. Utilizing the Spatial Variation in Temporal Trends (SVTT) technique, the study identifies municipalities’ vaccination trends. It also employs time series analysis and Interrupted Time Series methods to evaluate the pandemic’s impact on vaccination rates, using data from the PNI and the Information System on Live Births (SINASC). Results: Between January 2014 and December 2023, Brazil administered 25,902,207 doses of the BCG vaccine to children at birth, with 3911 municipalities (70.24%) showing declining trends, particularly in Florianópolis. Similarly, 22,962,434 doses of the Hepatitis B vaccine were administered, with 3284 municipalities also experiencing declines. Conclusions: It is crucial that public health policies be reevaluated to address regional disparities in vaccination coverage, particularly in more vulnerable areas. Focused interventions, such as awareness campaigns, improved access to vaccination services, and strengthened monitoring, are fundamental to reversing this trend.

## 1. Introduction

Vaccines represent one of the greatest achievements in the field of public health, having a substantial impact on child survival and the control and eradication of diseases worldwide [1]. Childhood immunization is widely recognized as one of the most effective interventions in public health for the prevention of infectious diseases, being a fundamental element in reducing child mortality [2]. In Brazil, the National Immunization Program (PNI) coordinates the development and operationalization of vaccination schedules, including the child vaccination schedule [3].

The PNI was implemented in 1973, expanding the distribution and regulation of immunobiologics, and has established itself as a successful program recognized internationally [4]. After its implementation, vaccination coverage significantly increased in the Brazilian population, especially among children at birth, whose vaccination coverage has remained above 95% since the 1990s, indicating high adherence from the population and the effectiveness of the program. However, since 2016, there has been a decline in coverage rates, exacerbated by measles outbreaks in Roraima and Amazonas, and more recently, by the COVID-19 pandemic in 2020 [5,6].

Currently, the childhood vaccination schedule includes 13 essential vaccines, including the Bacillus Calmette–Guérin (BCG) vaccine and the Hepatitis B vaccine, which are both recommended to be administered at birth. These immunobiologics provide early protection against tuberculosis and hepatitis [7,8,9]. The BCG vaccine is widely recognized for its effectiveness in preventing severe forms of tuberculosis, particularly meningeal and miliary forms, which are among the leading causes of mortality from the disease [7]. The Hepatitis B vaccine is important for preventing vertical transmission of the virus, which is particularly dangerous during the neonatal period, significantly reducing the risk of chronic infections and their long-term complications [10].

For more than two decades, Brazil had been achieving relevant vaccination coverage for these vaccines, especially at birth, thanks to the robust structure of the PNI. However, since 2015, with a marked intensification during the COVID-19 pandemic, there has been concern regarding the reduction of vaccination coverage in Brazil and in other countries [11,12]. Restrictive measures and the redirecting of health services to combat the SARS-CoV-2 virus hindered access to vaccination services, resulting in the interruption of immunization campaigns in several Brazilian municipalities [5,13,14].

Due to the distancing measures established during the pandemic, in-person attendance at health services decreased significantly, including for childhood vaccination. Countries like the USA, England, and Indonesia experienced considerable declines in vaccination rates among children at birth right at the beginning of the emergency declaration due to difficulties in accessing vaccination services, parents’ choices to delay vaccinations, confusion over the continuity of routine vaccinations, and even pre-existing inequalities in acceptance of childhood vaccination [15,16,17,18].

In this context, the World Health Organization (WHO) [19] established the Immunization Agenda for 2030, advocating for a comprehensive response to the COVID-19 pandemic through the rebuilding of childhood and adult immunization programs, including for COVID-19. It is worth noting that childhood vaccination offers protection against a range of infectious diseases, including measles, mumps, rubella, poliomyelitis, diphtheria, tetanus, pertussis, Hepatitis B, chickenpox, and rotavirus, among others [17].

Last decade, vaccination coverage for BCG and Hepatitis B in Brazil experienced significant declines, reflecting challenges in achieving immunization targets. For BCG, coverage dropped from 105% in 2015 to 77% in 2021, with a partial recovery to 90% in 2022 [20,21]. Hepatitis B vaccination declined from 90% in 2015 to 65% in 2020, before increasing to 82% in 2022 [20,22]. These fluctuations were exacerbated during the COVID-19 pandemic, underscoring the need for effective strategies to restore optimal vaccination levels.

Given this, it is necessary to investigate how such variations in vaccination coverage have behaved over the last decade through the analysis of time series data on BCG and Hepatitis B vaccination coverage. Thus, the present study aims to identify areas with spatial variation in the temporal trend of BCG and Hepatitis B vaccination coverage at birth, classify the identified temporal trends, and investigate the impact of COVID-19 on vaccination adherence.

## 2. Materials and Methods

### 2.1. Study Design and Setting

This is an ecological study [23,24] using real-world data conducted in Brazil, located in South America, with a territorial extent of 8,515,767 km^2^ and an estimated population of 208.4 million inhabitants. The ecological unit of analysis used in the study was the 5568 municipalities that comprise the country, distributed across 26 federal states (in addition to the Federal District) and five macro-regions (north, northeast, south, southeast, and central-west) [25].

### 2.2. Study Population and Source of Information

The study population consisted of the total number of doses of the immunizers, Hepatitis B and BCG, administered between 2014 and 2023 in children at birth. Data regarding vaccination and administered doses were obtained through the National Immunization Program (PNI) via DataSUS, and the population of interest was obtained from the Live Birth Information System (SINASC).

### 2.3. Spatial Variation in Temporal Trends—SVTT

To detect areas with a decreasing trend in vaccination, a spatial analysis technique called Spatial Variation in Temporal Trends (SVTT) was employed, developed by Kulldorff and Nagarwalla (1995) [26]. This technique differs from commonly used scanning analyses (purely spatial and space-time) because it calculates the temporal trends of clusters and compares them with other analyzed areas [27].

The identification of clusters occurs through the positioning of a circle with a variable radius around the centroid of each analysis unit (municipalities), calculating the number of observed and expected cases. This procedure is performed for all centroids, and when the observed value within the circle is significantly greater or lesser than expected, it is considered a cluster [28]. It is essential to highlight that SVTT does not aim to find clusters with a high or low number of occurrences of the event but rather to determine whether the temporal trend of that event is increasing or decreasing over time [28].

Therefore, in SVTT, the null hypothesis is that there is no difference in temporal trends in the analyzed areas, and the alternative hypothesis considers that the temporal trends are distinct. Temporal trends are calculated both within and outside the scanning circle. The change in the temporal trend of the analyzed event within a cluster is called an Internal Temporal Trend (ITT), while the trend of all other areas not belonging to that cluster is called an External Temporal Trend (ETT). Thus, the statistically significant aspect of this analysis is the temporal trends, not the formation of the cluster itself, as occurs in purely spatial and/or space-time scanning [27,29].

The parameters adopted for the analysis were as follows: discrete Poisson model, absence of geographic overlap of clusters, circular-shaped clusters, and 999 replications in Monte Carlo simulation; the exposed population was set at 3%, a value determined by the Gini coefficient, which compares the number of cases to the base population data; and the expected number of cases in each municipality was proportional to the size of the population at risk [28,30].

The analyses were conducted using SaTScan software version 9.3, and thematic maps were created using ArcGIS software version 10.5.

### 2.4. Time Series Analysis

Subsequently, monthly time series of vaccination coverage rates were constructed. To calculate vaccination coverage, the numerator considered the number of doses of each immunizer of interest administered in Brazil, and the denominator considered the population of live births that year, multiplying the result by the constant 100 to obtain the percentage of vaccinated children at birth.

To investigate the temporal pattern of vaccination coverage throughout the study period and identify its temporal trend, the Seasonal Trend Decomposition using Loess (STL) method was used, based on locally weighted regression [31]. The analysis was conducted in RStudio, using the forecast package [32].

Additionally, the Prais–Winsten autoregression method was performed using STATA version 14 to classify the temporal trend of vaccination coverage as increasing, decreasing, or stationary during the study period. For cases where the temporal trend was classified as increasing or decreasing, the monthly percent change (MPC) was calculated along with its respective 95% confidence intervals (CI95) [33].

To verify the impact that the COVID-19 pandemic had on these indices, the Interrupted Time Series technique was applied, which is described as the most effective resource for assessing the impact of an intervention, with two parameters defining each segment of the series: level and trend [34]. The level is considered the initial value of the series in each segment, and the trend is the percentage change in values over the period covered by the segment [33].

The goal was to assess whether, when an intervention occurs, there is an immediate impact (level change) and/or progressive impact (trend change) on the values of the series [34]. The software used for this analysis was also STATA version 14.

In the present study, we considered the “intervention” as the level and “post-intervention” as the progressive impact caused by the COVID-19 pandemic. The cutoff point considered in the study was February 2020 when the first case of the new coronavirus was diagnosed and confirmed in the country [35].

### 2.5. Ethical Aspects

The study utilizes publicly accessible data, with aggregated information and without individual identification, and, therefore, does not require evaluation and approval by a Research Ethics Committee (CEP).

## 3. Results

**Figure 1:**
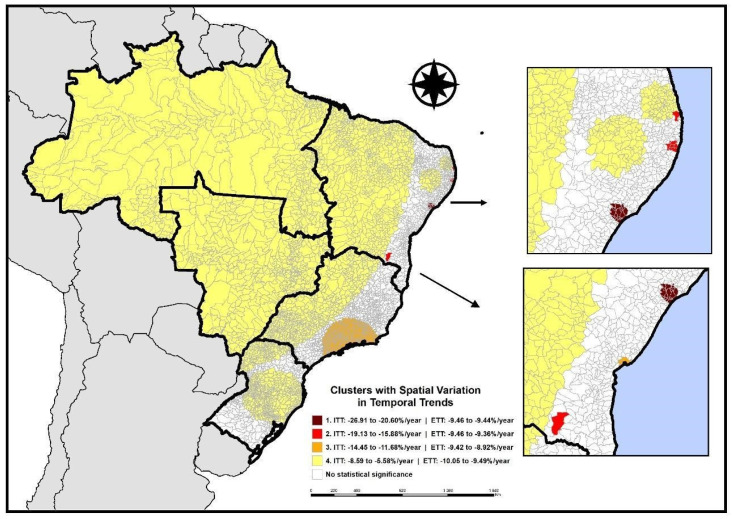
Areas with spatial variation in the temporal trend of BCG vaccination at birth, Brazil (2014–2023).

Between January 2014 and December 2023, Brazil recorded a total of 25,902,207 doses of the BCG vaccine administered to children at birth. Figure 1 presents the areas with spatial variation in temporal trends in BCG vaccination coverage, where 3911 municipalities (70.24% of Brazil) showed a decline in the Temporal Trend Index (TTI) across the four identified clusters. Among the 22 municipalities that make up the cluster with the greatest decrease, Florianópolis (in the state of Santa Catarina, southern region of the country) stands out as the municipality with the highest drop in BCG vaccination coverage (TTI: −26.6%/year), followed by 21 municipalities in the state of Sergipe (northeastern region of the country) with a TTI of −20.60%/year.

The second cluster with the greatest decrease in BCG vaccination coverage (TTI: −19.13 to −15.88%/year) includes seven municipalities in Pernambuco (northeastern region of Brazil) and three municipalities distributed among Bahia, Paraíba, and Ceará, which are all located in the northeastern region of Brazil. The third cluster, composed of 395 municipalities (TTI: −14.45 to −11.68%/year), is distributed among Minas Gerais (224 municipalities in the southeastern region), São Paulo (96 municipalities in the southeastern region), Rio de Janeiro (70 municipalities in the southeastern region), and Bahia (5 municipalities in the northeastern region). Another 3484 municipalities that showed a reduction in BCG vaccination coverage (TTI: −8.59% to −5.58%/year) are scattered across all regions, with states in the northern and central-western regions entirely encompassed in this last cluster. Espírito Santo (southeast) and Alagoas (northeast) did not show a significant decrease in vaccination coverage (Figure 1).

**Figure 2:**
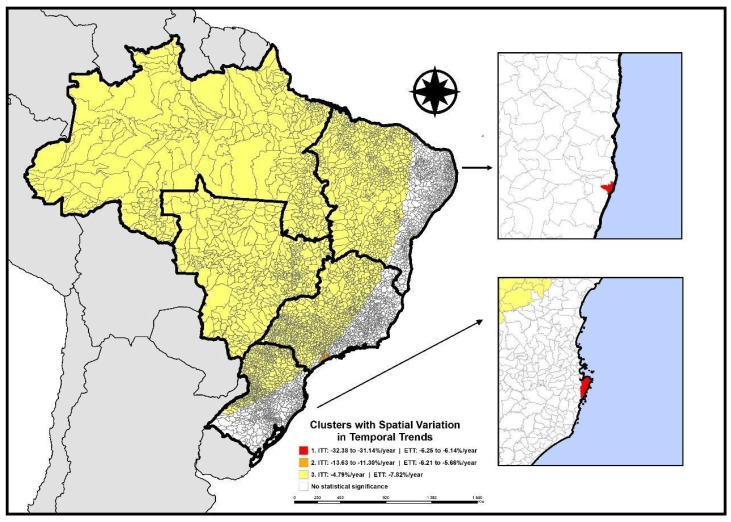
Areas with spatial variation in the temporal trend of Hepatitis B vaccination at birth, Brazil (2014–2023).

Brazil recorded a total of 22,962,434 doses of the Hepatitis B vaccine administered to children at birth during the analyzed period. Through application of the SVTT technique, it was found that 3284 (59.0%) Brazilian municipalities exhibited spatial variation in the temporal trend in Hepatitis B vaccination coverage, according to Figure 2, with a decline in the Temporal Trend Index (TTI) across the three identified clusters. The municipalities of Olinda (Pernambuco, northeastern region) and Florianópolis (Santa Catarina, southern region) comprise the cluster with the greatest decrease in TTI (−32.4%/year and −31.1%/year, respectively), while the cluster with the second greatest decrease in vaccination coverage (TTI: −13.63 to −11.30%/year) includes 27 municipalities, with 26 located in the state of São Paulo (southeastern region) and one in Ceará (northeastern region). The third cluster, with a TTI of −4.79%/year, is composed of 3250 municipalities distributed across the northern (450 municipalities), northeastern (764 municipalities), central-western (467 municipalities), southeastern (983 municipalities), and southern (586 municipalities) regions of Brazil (Figure 2).

**Figure 3:**
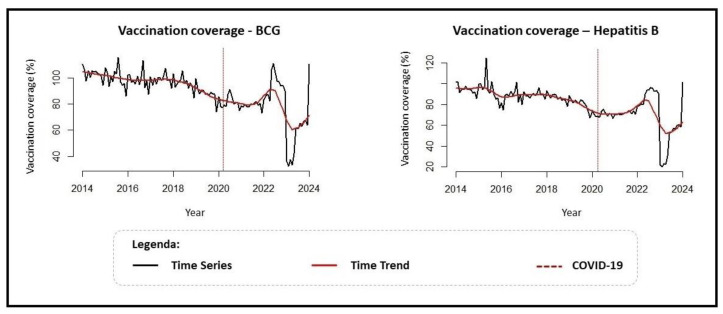
Historical series and temporal trend of vaccination coverage for BCG and Hepatitis B at birth, Brazil (2014–2023).

Figure 3 presents a time series (historical series) of vaccination coverage for the analyzed immunizers and the estimated temporal trend. We can observe that the coverage for BCG and Hepatitis B follows similar patterns, with a slight decrease in the temporal trend until 2021 when there is a growth in 2022, followed by another decline, which this time is more pronounced, in 2023.

The temporal trend (estimated and presented graphically in Figure 3) was classified using the Prais–Winsten autoregression technique as shown in Table 1 below. The temporal trend of BCG vaccination coverage in children at birth was classified as decreasing, with a decline of 0.43% per year (95% CI = −0.66 to −0.19). The coverage for Hepatitis B was also classified as decreasing, with a decline of 0.49% per year (95% CI = −0.81 to −0.16), corroborating the findings presented in Figure 1.

Regarding the Interrupted Time Series (ITS), used for the purpose of verifying the impact caused by the COVID-19 pandemic, no level change was observed, meaning there was no abrupt decline in the temporal trend of vaccination coverage with the emergence of COVID-19; however, a progressive change was noted in the temporal trend of BCG vaccination coverage, where the decreasing trend intensified, showing a decline of −0.81% per year after February 2020 (95% CI = −1.50 to −0.09) according to Table 1.

## 4. Discussion

The study aimed to identify areas with spatial variation in the temporal trend of BCG and Hepatitis B vaccination coverage in children at birth, classify the identified temporal trends, and investigate the impact of COVID-19 on vaccination adherence. The analyses allowed for identification of heterogeneity among Brazilian municipalities regarding BCG and Hepatitis B vaccination coverage. The Brazilian National Immunization Program (PNI) is one of the most comprehensive immunization programs in the world, recognized for the collective and individual strategies that ensured high vaccination coverage for almost all immunobiologicals over several decades, which allowed for a reduction in incidence rates and deaths from vaccine-preventable diseases [36,37,38]. However, the decrease in vaccination coverage rates in recent years invites us to reflect on the fragility of collective immunization and the risk of resurgence of diseases that were previously controlled or eradicated [39].

The findings of this study indicate a significant decline in vaccination coverage for essential vaccines in children to protect them in their early days of life, especially after the onset of the COVID-19 pandemic. Between 2014 and 2023, regional and temporal variations in the vaccination coverage trends for BCG and Hepatitis B were observed, suggesting differentiated impacts across various regions of the country. This scenario is consistent with global evidence indicating a decrease in vaccination coverage during the pandemic, resulting from multiple factors, such as interruptions in health services, vaccine hesitancy, changes in immunization policies, and prioritization of pandemic responses [39,40,41].

In this direction, studies show that difficulties in accessing vaccination represent one of the barriers to increasing vaccination coverage in many countries, with 20 million children unvaccinated annually, particularly among the most vulnerable and those living in conflict-affected countries or remote areas [37]. On the other hand, positive perceptions about the safety and efficacy of vaccines have decreased over the years, even in countries with high levels of education and adequate access to health services, compounded by the healthcare system’s overload due to the COVID-19 pandemic and the numerous fake news items spread by anti-vaccine groups worldwide [36,37,38].

The study highlights the BCG vaccine, indicating that the city of Florianópolis (south region) along with 21 municipalities in Sergipe (northeast region) experienced the largest declines in coverage. This distribution suggests significant regional inequalities. In the international context, Dhamania and Gaur (2021) [42] identified declines in immunization during the pandemic in India, which correlated with reductions in health services and temporary closures due to the pandemic. Globally, 25 million children were either unvaccinated or under-vaccinated by 2020, marking the largest sustained decline in childhood vaccinations in the past 30 years. The WHO Southeast Asian Region was the most affected, with a 9% drop in essential immunization service coverage in the first 2 years of the pandemic [42].

BCG vaccination coverage in Brazil has shown a declining trend since 2016, mainly due to difficulties in production and distribution at the national level, leading to a gradual decrease in coverage, which worsened with the COVID-19 pandemic [43,44,45]. In the case of the Hepatitis B vaccine, production is carried out by the Butantan Institute, ensuring availability in the Unified Health System (SUS) and the continuity of immunization actions [46,47,48]. The Hepatitis B vaccine is essential to prevent vertical transmission of the virus, which can occur during the neonatal period and is associated with high rates of chronic infection and severe long-term complications, such as cirrhosis and hepatocellular carcinoma [39]. These conditions highlight the importance of maintaining regular production and high coverage of these vaccines to protect vulnerable groups and reduce the impact of preventable diseases.

The declines in vaccination coverage for BCG and Hepatitis B vaccines in Brazil indicate significant fragilities in monitoring childcare, especially regarding the initiation of immunization in maternity wards. The BCG vaccine, aimed at preventing severe tuberculosis, has already shown a continuous decline since 2016, partly due to the lack of national production, and in 2016, only 44% of municipalities reached the recommended coverage for this vaccine [43,44,45]. On the other hand, despite the Hepatitis B vaccine being administered in maternity wards and produced by the Butantan Institute, its coverage also fell, reflecting the urgent need to enhance vaccination strategies and ensure the standardization and continuity of vaccination [49].

The relationship between temporal and spatial trends observed in the study reveals the complexity of the factors that influence vaccination rates. We observed that the decline in vaccination rates in certain regions over time may be associated with a combination of external factors, such as public health policies, accessibility to vaccination services, and levels of vaccine hesitancy [50]. For example, regions with lower access to health centers or with less intensive vaccination campaigns tend to show a sharper decline in vaccination rates. In addition, vaccine hesitancy, influenced by cultural and socioeconomic factors, can vary significantly across different regions, affecting both temporal and spatial trends [51].

Evidence that the decline in vaccination coverage reflects vulnerabilities in neonatal health practices and childcare management is supported by data showing heterogeneity in vaccination rates across different regions of Brazil [45]. This underscores the importance of reevaluating immunization strategies, ensuring that at birth babies consistently receive necessary vaccines, especially BCG, in the maternity environment to protect public health in the long term [43,45].

To improve vaccination coverage in vulnerable areas in Brazil, it is essential to adopt specific policies that consider the particularities of these regions. Implementing microplanning strategies, such as holding vaccination “D-Days” and actively seeking out unvaccinated individuals, can significantly increase vaccination coverage. These actions should be carried out not only in health facilities but also in schools, workplaces, and community centers [52]. In addition, improving health infrastructure in vulnerable areas is also essential, ensuring that vaccination centers are accessible and well-equipped, including providing free or subsidized transportation to facilitate access to vaccination services for more distant populations [53]. Developing education and awareness campaigns focused on vulnerable communities and groups, and using local languages and media also help increase vaccination coverage, since campaigns address the importance of vaccination, combat misinformation, and increase confidence in vaccines, especially when city governments establish partnerships with community organizations, NGOs, and local leaders to promote vaccination. These partnerships can help reach populations that traditionally have less access to health services and increase uptake of vaccination campaigns [54].

Implementing continuous monitoring and evaluation systems to identify areas with low vaccination coverage and adjust strategies as needed is essential, and this includes using geospatial data to map vaccination coverage and direct resources more effectively, so that studies using real-world data can help improve vaccination coverage in a more targeted and effective way, meeting the specific needs of vulnerable areas in Brazil.

The implementation of the National Immunization Program Information System (SI-PNI) has been a powerful tool to address gaps in vaccination data, but the reality of municipalities often complicates this integration, revealing the potentials and limitations of computerized systems concerning the assessment of vaccination coverage and surveillance of adverse events, highlighting the need for a joint effort to improve data quality and the effectiveness of vaccination campaigns [55]. Moura et al. (2024) [56] highlight the heterogeneity in vaccination indicators and the importance of spatial analysis to understand variations in vaccination coverage, which can be essential for formulating more effective public policies. These challenges underline the need for standardization and effective integration of systems that not only improve data collection but also facilitate decision-making in public health and the continuity of immunization actions [55].

Given the current situation, it is important to mention that the anti-vaccine movement has proven to be a serious threat to public health in Brazil, contributing to the drop in vaccination coverage rates in several regions of the country. Studies indicate that vaccine refusal, driven by misinformation and distrust of vaccines, has led to the resurgence of previously controlled diseases, such as measles and yellow fever [57]. This phenomenon is particularly worrying in vulnerable areas, usually related to socioeconomic and cultural factors that vary significantly between different locations, where the lack of access to accurate information and the influence of anti-vaccine groups can exacerbate vaccine hesitancy [58]. In areas with less access to health services and education, misinformation tends to spread more easily, resulting in lower vaccination rates. In addition, the influence of community leaders and the presence of anti-vaccine campaigns on social media have contributed to the dissemination of myths about the safety and efficacy of vaccines [59]. Therefore, it is crucial that public health policies address these issues head-on, promoting awareness and education campaigns to combat misinformation and increase confidence in vaccines.

As limitations of the study, the use of secondary data, while providing a broad and accessible view of the vaccination situation, may not capture the entirety of the local context and the nuances that influence vaccination rates. Therefore, it is crucial to conduct new studies that complement this data with primary studies involving field information collection, allowing for a more robust and contextualized analysis of vaccination dynamics.

Despite the advantages of using cluster identification analysis, it is important to recognize some inherent limitations of this approach. First, the assumption that clusters are perfectly circular may not accurately reflect actual geographic and demographic distributions, which often have irregular shapes. Furthermore, the lack of overlap between clusters may limit the ability to detect high-density areas that span multiple adjacent clusters. These limitations may result in an underestimation of the true extent of clusters and, consequently, a less accurate interpretation of spatial patterns. Therefore, future studies should consider incorporating more flexible cluster shapes and analytical methods adapted to geography and population distribution to improve the accuracy of the analyses.

Although studies suggest an association between declining vaccination rates and worsening of certain diseases, this relationship could not be inferred in this study. Specifically, the increase in childhood tuberculosis cases cannot be linked to the decline in BCG vaccination, nor can the decline in hepatitis B vaccination coverage be attributed to the increase in cases of the chronic disease in children at birth. This limitation is due to the lack of a longitudinal study, which would be necessary to understand the long-term effects of vaccination and to identify other multiple factors that may influence the prevalence of these diseases in childhood.

Finally, the results emphasize the need for more research to explore the factors associated with the decrease in vaccination coverage and the potential long-term impacts on public health, especially in scenarios of future health crises. These actions are essential to ensure the ongoing protection of the child population and to prevent outbreaks of vaccine-preventable and/or already eradicated diseases.

## 5. Conclusions

The findings of this study corroborate global evidence demonstrating the effects of the COVID-19 pandemic on the reduction of vaccination coverage. The regional and temporal analysis highlights the need for targeted actions to address inequalities and promote the recovery of immunization rates. Strategies that integrate regional and national measures are essential for restoring vaccination rates and preventing future public health crises, especially in a post-pandemic scenario. These efforts should involve a multifaceted approach, considering not only the logistical aspects of vaccination but also public trust and perceptions regarding the safety and efficacy of vaccines.

It is crucial that public health policies be reevaluated to address regional disparities in vaccination coverage, particularly in more vulnerable areas. Focused interventions, such as awareness campaigns, improved access to vaccination services, and strengthened monitoring, are fundamental to reversing this trend. Additionally, it is necessary to consider the development of new strategies for immunization and protection of the most affected populations, including the use of new monitoring technologies and evidence-based strategies to maximize the effectiveness of immunization programs.

## Figures and Tables

**Table 1:** Classification of the temporal trend of vaccination coverage for BCG and Hepatitis B in children at birth, Brazil (2014–2023).

**Time** **Series Analysis (Prais–Winsten)**			
Immunizer	Coefficient (95% CI)	Trend	MPC (95% CI)
**BCG**	−0.001 (−0.002; <−0.000)	Descending	−0.43% (−0.66; −0.19)
**Hepatitis B**	−0.002 (−0.003; <−0.000)	Descending	−0.49% (−0.81; −0.16)
**Interrupted Time Series** **Intervention: COVID-19 (Cutoff Point: Feb 2020)**			
Immunizer	Coefficient (95% CI)	Trend	MPC (95% CI)
**BCG**	−0.033 (−0.116; 0.049)	Stationary	-
**Hepatitis B**	−0.026 (−0.140; 0.086)	Stationary	-
**Interrupted Time Series** **Post-Intervention: Post-COVID-19**			
Immunizer	Coefficient (95% CI)	Trend	MPC (95% CI)
**BCG**	−0.03 (−0.006; <−0.000)	Descending	−0.81% (−23.60; −0.09)
**Hepatitis B**	−0.004 (−0.008; 0.001)	Stationary	-

Source: author’s own.

## Data Availability

All data used in the study are in the public domain and available on DATASUS Tabnet.
